# Psychometric Properties of the Decisional Balance Scale: A Confirmatory Study on Malaysian University Students

**DOI:** 10.3390/ijerph17082748

**Published:** 2020-04-16

**Authors:** Kien Ting Liu, Yee Cheng Kueh, Wan Nor Arifin, Mohd Ismail Ibrahim, Mohd Nazri Shafei, Garry Kuan

**Affiliations:** 1 Unit of Biostatistics and Research Methodology, School of Medical Sciences, Universiti Sains Malaysia, Kubang Kerian 16150, Kelantan, Malaysia; kienting@hotmail.com (K.T.L.); wnarifin@usm.my (W.N.A.); 2 National Heart Association of Malaysia, Kuala Lumpur 59200, Malaysia; 3 Department of Community Medicine, School of Medical Sciences, Universiti Sains Malaysia, Kubang Kerian 16150, Malaysia; ismaildr@usm.my (M.I.I.); drnazri@usm.my (M.N.S.); 4 Exercise and Sports Science Programme, School of Health Sciences, Universiti Sains Malaysia, Kubang Kerian 16150, Kelantan, Malaysia; 5 Department of Life Sciences, Brunel University, London UB8 3PH, UK

**Keywords:** reliability, factor analysis, psychometric, university, student, exercise

## Abstract

Decisional balance (DB) is the perceived positive aspects (advantages) and negative aspects (disadvantages) that are associated with behavioural change. Behavioural change is dependent on an individual’s thoughts after considering the advantages of engaging in exercise. When the benefits exceed the barriers, people are more likely to make changes after cognitively evaluating the functional aspects. The purpose of the present study is to determine the validity and reliability of the DB scale among Malaysian university students using a confirmatory factor analysis (CFA). A cross-sectional study was carried out among students who took part in the co-curricular program. By using the purposive sampling method, students were recruited and given written informed consent forms after acknowledging they understood the purpose of the study. The DB scale, which consists of two factors, namely, advantages and disadvantages, was used as the instrument in the study. The advantages referred to the benefits of participating in exercise, whereas the disadvantages referred to the barriers to exercise. The 10-item, self-administered questionnaires were distributed to participating students. Data were analysed using Mplus 8 for the CFA. A total of 562 students (females = 444, males = 118) with a mean age of 19.81 years (*SD* = 1.22) participated in the study. Most of the students were engaged in regular physical activity for at least three exercise sessions (mean = 2.62) per week, and the average duration per session was 43 minutes. The hypothesised measurement model of DB did not fit the data well; thus, the measurement model was re-specified. The final measurement model fit the data well (comparative fit index (CFI) = 0.960, Tucker–Lewis index (TLI) = 0.943, standardised root mean square residual (SRMR) = 0.055, root mean square error of approximation (RMSEA) (90% confidence interval (CI)) = 0.061 (0.047, 0.074), RMSEA *p*-value = 0.096). The composite reliability values of 0.757 for the advantages and 0.792 for the disadvantages were acceptable. The 10-item DB scale with two factors displayed a good model fit for the data with good scale reliability. This could be beneficial for Malaysian undergraduate students in making decisions before engaging in physical activity. The benefits of, and barriers to, exercise could be an important component that affects their decision making.

## 1. Introduction

Physical inactivity is recognised as the fourth leading mortality risk factor, which causes 6% of deaths worldwide [1]. Studies have shown that large parts of the population in the United States [2], Europe [3] and Malaysia [4] do not actively participate in regular physical activity (PA) despite its benefits [5].

To encourage individuals to engage in PA, the transtheoretical model (TTM) was introduced and used widely to reveal how an individual’s behavioural change can be viewed as a process involving progression through a series of stages (pre-contemplation stage, contemplation stage, preparation stage, action stage and maintenance stage) [6]. The TTM consists of stages of readiness for change, processes of change, advantages and disadvantages of changing and self-efficacy that could help individuals switch to new behaviour through a series of changes [7]. Upon adopting a new behaviour, it is most important that a person has the right attitude and positive thoughts regarding the benefits of exercise. People tend to change exercise habits themselves after considering the advantages and disadvantages of the activity. Decision making could influence them to think of the possible options and evaluate the consequences of each option before changing their exercise habits. Some will consider the costs compared to the advantages when deciding whether to change or not [8]. Thus, by weighing the advantages and disadvantages from time to time, they will decide on the best strategies for themselves. 

Decisional balance (DB) is the perceived positive aspects (advantages) and negative aspects (disadvantages) that are associated with behavioural change [9]. The two components of DB, the advantages and disadvantages, were taken from the decision-making model developed by Janis and Mann [10], and the perception of positive and negative aspects is related to an individual’s behavioural changes. Examples of advantages in exercise were improved aerobic capacity, self-esteem and muscular strength [11], whereas examples of disadvantages included physical discomfort, cost and taking time away from other activities [11]. Within this context, the DB scale was used to determine the strategies employed by university students in perceiving the advantages and disadvantages of exercise adoption to enhance their PA levels [12]. 

According to individuals who successfully changed their behaviour, they noticed the advantages of the behavioural change more than the disadvantages, and the advantages of exercise outweighed the disadvantages [8]. Typically, people tend to have positive views and beliefs in the early stages and negative views and beliefs in the later stages. DB was linked with the stages of change, and it was also theorised that DB would increase from pre-contemplation to maintenance [13,14], which has become an important construct in the TTM.

According to Han, Gabriel and Kohl [15], the TTM indicated that people begin to perceive more benefits than disadvantages in adopting positive behavioural changes as they move through the later stages. This statement was also supported by Prochaska and Velicer [13], who stated that the disadvantages outweigh advantages in the pre-contemplation stage; however, the advantages appear to be at the same level as disadvantages in the contemplation or preparation stages. A new behaviour is established and becomes part of one’s lifestyle when the advantages outweigh the disadvantages in the action and maintenance stages. A study conducted by Zamarripa et al. [16] found that self-determined motivation is significantly related to the prediction of exercise enjoyment by decision balance. Thus, the study supports the statement of Han et al. [15] by showing that the advantages of exercise are linked to behavioural changes as the motivation increases.

Students were apt to be recruited for the present study because previous research demonstrated that they differ from college drop-outs in terms of the psychological and environmental factors associated with exercise adoption and maintenance [17]. Therefore, studying their exercise behaviour in terms of DB in exercise is crucial. Moreover, there were a limited number of published studies on the validation of the DB scale. Previous researchers reportedvalidation results based on principal component analysis [18] and the stability of the construct across time based on a longitudinal study [19]. In addition, Malaysia is a multi-racial country with diverse cultures. This may prompt a different perspective related to exercise behaviour, which could possibly produce different structural factors when a Western developed instrument, such as the DB scale, is applied to a Malaysian population. Furthermore, there were limited valid instruments to measure the exercise DB among Malaysian populations. A recent study conducted by Rizal et al. [20] employed the translated Malay version of DB among Malay students aged 10–12 years, which showed only an adequate fit on the DB scale and fell slightly below the cut-off point, set at 0.90 for comparative fit index (CFI) and Tucker–Lewis index (TLI). Thus, the present study was conducted to determine the validity and reliability of the DB scale among Malaysian university students, who are older (>18 years old), using a confirmatory approach. 

## 2. Materials and Methods 

### 2.1. Procedures and Study Settings

A cross-sectional study was conducted from 29 October 2017 to 30 April 2018 at the Health Campus, Universiti Sains Malaysia (USM). Using the self-administered method, the questionnaire was distributed to undergraduate students according to their co-curricular programs. By using a purposive sampling method, those who enrolled in co-curricular programs, were available and agreed to participate during data collection were recruited as participants. The sampling method was used to ensure that the study had adequate participants from all exercise levels to represent the scale, thus making the scale valid and reliable. Besides, the sampling method could reduce the floor and ceiling effects of participants’ responses on the scale. An informed consent form and a set of questionnaires were provided after the participants understood the purpose of the study. 

The study was approved by the Human Research Ethics Committee of Universiti Sains Malaysia (USM/JEPeM/17070322), and it followed the guidelines of the International Declaration of Helsinki. During the data collection phase, a research information form was given to all participants to ensure they understood the study. The research information sheet included the study purpose, procedures, and potential risks and benefits. They were informed that their participation was entirely voluntary, and they were free to withdraw at any time. Finally, the written informed consent was obtained from the potential participants.

### 2.2. Participants

Undergraduate students who took part in the co-curricular programs in the first semester of the 2018/2019 academic year were recruited as study participants. The undergraduates were comprised of students in their first, subsequent, or final years of studies who participated in sports, art and uniform programs. Out of 600 distributed questionnaires, 562 participants completed and returned the forms, yielding a response rate of 93.7%. The participants were composed of 118 males (21.0%) and 444 females (79.0%). The mean age of the participants was 19.81 (1.22) years, with ages ranging from 18 to 27 years. Most of the students were first-year undergraduates from the School of Health Sciences (67.3%) and engaged in regular physical activity for at least three exercise sessions (mean = 2.62) per week, with an average time per session of 43 minutes. Kline [21] suggests that a reasonable sample size for studies using structural equation modelling (SEM) is about 200 cases. Confirmatory factor analysis (CFA) is one of the components of SEM. Thus, the present sample size of 600 undergraduate university students was deemed adequate.

### 2.3. Instrument

The 10- item Decisional Balance scale, which originated with Plotnikoff et al. [19], was used in the study. Participants were asked to indicate their response (e.g., “PA would help me reduce tension or manage stress”) using a 5-point Likert scale ranging from 1 “not at all confident” to 5 “extremely confident”. The DB scale with two factors, Pros (advantages in exercise) and Cons (disadvantages in exercise), represented the individual’s positive and negative views towards exercise. The internal consistency reliability was reported to be 0.82 for Pros and 0.72 for Cons [19].

### 2.4. Statistical Analysis

Statistical analysis was conducted using Mplus version 8. Continuous variables were presented as mean and standard deviation (*SD*), while categorical variables were tabulated using frequency and percentage in the descriptive analysis. By using the robust maximum likelihood estimator (MLR), a confirmatory factor analysis (CFA) was performed to assess the psychometric properties of the DB scale. Several fit indices were used to evaluate and compare the model fitness as recommended by Hair et al. [22]: standardised root mean square residual (SRMR) of less than 0.08 with a *p*-value of less than 0.05, root mean square error of approximation (RMSEA) of less than 0.05, comparative fit index (CFI) of more than 0.95 and Tucker–Lewis index (TLI) of more than 0.95. By rule of thumb, a good standardised loading factor of each measurement latent variable, which is quantified from the manifest variable, should be above 0.5 and ideally 0.7 or higher [22]. The initial model was tested to see the model fitness based on the above fit indices. The specification was made by evaluating the acceptability of the model and correlating the item’s residual based on adequate theoretical support. The items were correlated after considering the meaning of the items and those that were within the same factor. In this study, a standardised item loading of 0.5 and above was used as the cut-off point. The internal consistency reliability of each factor was estimated using composite reliability based on CFA results. A reliability coefficient of more than 0.70 was considered as adequate [21]. For the discriminant validity, a correlation *r* between factors less than 0.85 indicated that the discriminant validity was established [21]. This was meant to ensure that no high correlation between two different factors would cause the model to have poor discriminant validity.

## 3. Results

**Figure 1:**
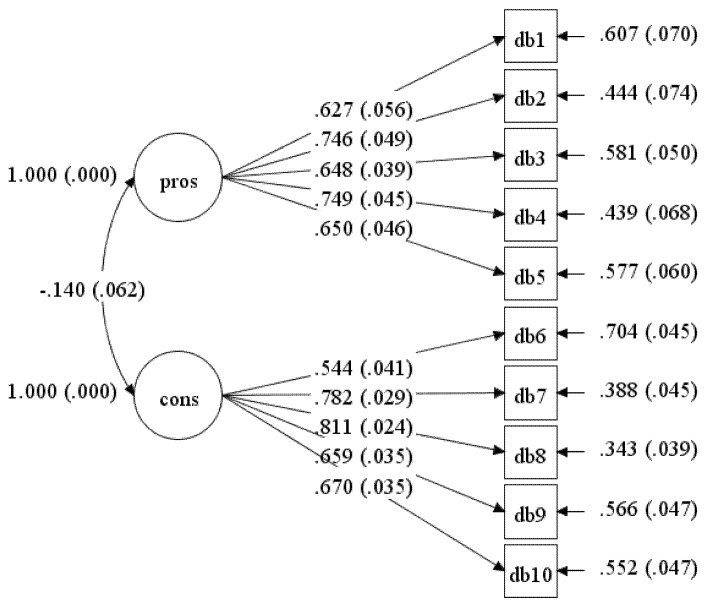
Standardised item loading for Model-1.

The initial hypothesised measurement model (Model-1) consists of two latent variables (factors) with ten observed variables (items). The result displayed a poor fit of data in Model-1 (see Table 1). Although the loading of all items was above 0.4 (see Figure 1), poor fit indices indicated the model did not fit the data well. 

**Figure 2:**
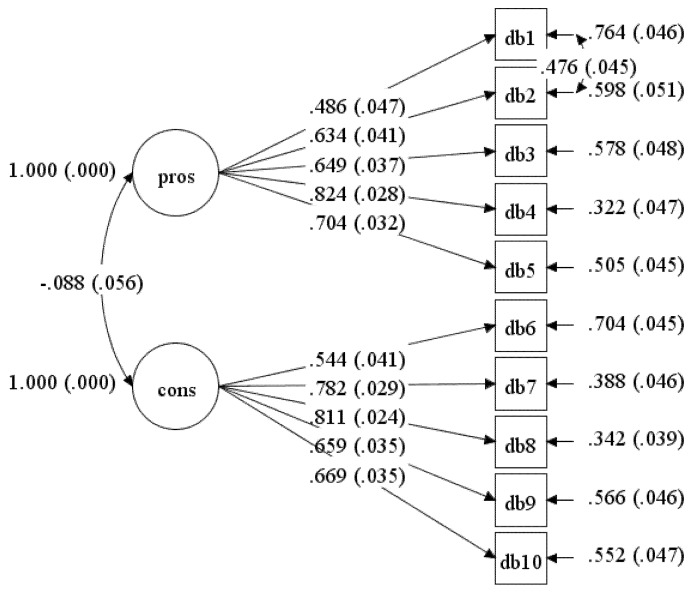
Standardised item loading for Model-2.

Further investigation was made beyond the initial model by correlating the residual for item DB2 with DB1 in Model-2. Analysis of this model indicated an improvement in Model-2 (see Table 1). However, only the fit indices of SRMR were within the recommended values. All items’ factor loadings in Model-2 were above 0.4 (see Figure 2). 

**Figure 3:**
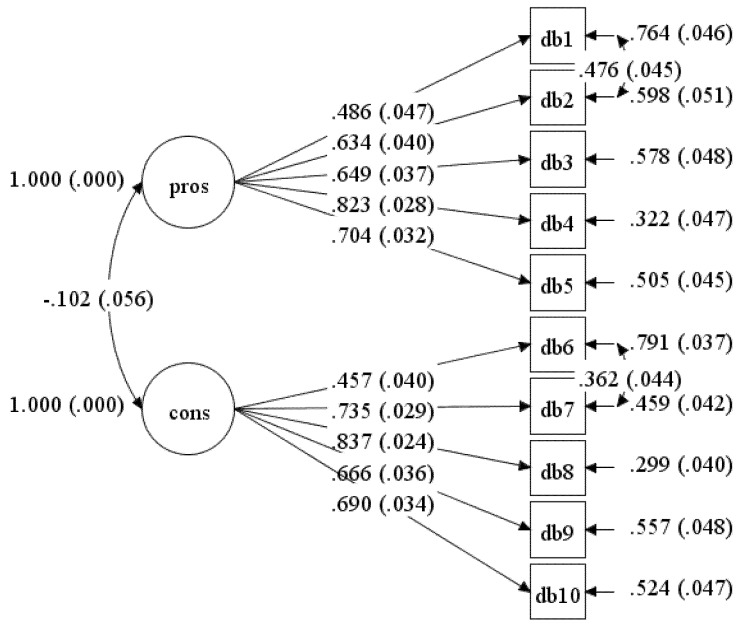
Standardised item loading for Model-3.

Model-2 was re-specified to improve the fit indices by correlating the residual for item DB2 with DB1 and DB7 with DB6 simultaneously (Model-3). Model-3 fit the data well and all the fit indices were within the recommended values (see Table 1). The final model, Model-3, consists of good and acceptable standardised item loading, which ranged from 0.486 to 0.824 (see Figure 3). 

The composite reliability value for Pros was 0.757 (95%CI: 0.716–0.799), whereas for Cons it was 0.792 (95%CI: 0.761–0.824). Both factors showed good construct reliability. There was a non-significant linear correlation between Cons and Pros with *r* = −0.102 (*p* = 0.066) among undergraduate students. This indicated a negative linear relationship and little correlation [23]. Since the *r* value was less than 0.85, the discriminant validity between the two factors was achieved.

## 4. Discussion

This study was designed to determine the psychometric properties of the DB scale when applied to undergraduate university students at the Health Campus, USM. The confirmatory analyses of ten items showed good fit indices and factor loadings for the model tested among undergraduate students. The initial model with ten items did not achieve the model fitness. Hence, re-specification was made based on high modification indices by correlating the residual for item DB2: “I would feel more confident about my health by doing PA” with item DB1: “Physical activity would help me reduce tension or manage stress”, as well as item DB7: “Physical activity would take too much of my time” with item DB6: “I am too tired to engage in physical activity because of my other daily responsibilities”. Based on theoretical support by Plotnikoff et al. [19], items DB1 and DB2 belong to the same advantages latent variable, while items DB6 and DB7 belong to the disadvantages latent variable. Hence, the inclusion of the correlated residuals was theoretically and statistically acceptable. 

This result showed that the DB scale is important for an individual’s behaviour modification. Their perception of the benefits and disadvantages of exercise influenced their health-related behaviour through the stages of change. The concept is similar to the TTM, where people in the early stages of change had a negative DB and progressively changed their attitudes to being more positive in the later stages [13]. They began to make changes after careful consideration of all aspects without counting any potential loss. The positive influence on physical activity behaviour could act as a motivation to encourage students to adopt positive lifestyle modifications. They could use the scale as a guideline to overcome obstacles during exercise and use different strategies accordingly [24]. Besides, successful people could achieve or accomplish their goals with positive thought [25]. This scale could be useful for coaches, sport psychologists and exercise educators as a guideline for observing an athlete’s performance. Athletes can also use the scale to evaluate their own energy levels to boost their performance. This could be useful for the development of Malaysia’s sport industry. Thus, the model plays an important role in determining individuals’ health-related behaviour in PA.

The standardised factor loadings of the three models were above or close to the recommended cut-off value identified by Hair et al. [22]. These models indicated that the items were able to represent their respective models. In the current study, composite reliability values of advantages and disadvantages were considered adequate as reported by Kline [26]. The findings are similar to an American study in 2012, which reported that the Cronbach’s alphas of advantages and disadvantages were 0.90 and 0.67, respectively [27]. Additionally, a study by Plotnikoff and colleagues [18], which tested the scale among Canadian adults, reported high composite reliability (0.82 for advantages and 0.72 for disadvantages).

Several limitations were encountered in this study. This study had an inherent limitation in measuring the students’ decision making related to their behaviour during PA. The self-administered method may introduce some biases, such as response bias and recall bias, which may affect the reliability of the scale. Thus, researchers encouraged every student to answer the questions sincerely and avoid discussing the questions with their friends.

## 5. Conclusions

The 10-item DB scale with two factors displayed a good model fit for the data with good scale reliability. This could be beneficial for Malaysian undergraduate students when making decisions before engaging in PA. The benefits of, and barriers to, exercise could be the vital components that affect their decision making. It can also be used as a guideline to help understand people’s thoughts in deciding to engage in PA. In addition, the DB scale would be an appropriate instrument for research and is applicable worldwide. 

## Figures and Tables

**Table 1:** Model fit indices for three decisional balance (DB) models.

Model	CFI	TLI	SRMR	RMSEA (90%CI)	RMSEA *p*-Value
Model-1 (Initial)	0.867	0.823	0.063	0.107 (0.095, 0.119)	<0.001
Model-2 ᵅ	0.929	0.903	0.060	0.079 (0.066, 0.092)	<0.001
Model-3 ^b^	0.960	0.943	0.055	0.061 (0.047, 0.074)	0.096

CFI = comparative fit index, TLI = Tucker–Lewis index, SRMR = standardised root mean square residual, RMSEA = root mean square error of approximation, CI = confidence interval, Cl fit = close fit. ᵅ Correlated residual for item DB2 with DB1. ^b^ Correlated residual for item DB2 with DB1 and DB7 with DB6.

## References

[1] WHO (2013). Global Strategy on Diet, Physical Activity and Health.

[2] Haskell W.L., Lee I.M., Pate R.R., Powell K.E., Blair S.N., Franklin B.A. (2007). Physical activity and public health: Updated recommendation for adults from the American college of sports medicine and the American heart association. Circ.

[3] Commission E. (2010). Sport and Physical Activity.

[4] Poh B.K., Safiah M.Y., Tahir A., Siti Haslinda N., Siti Norazlin N., Norimah A.K. (2010). Physical activity pattern and energy expenditure of Malaysian adults: Findings from the Malaysian Adult Nutrition Survey (MANS). Malays J. Nutr..

[5] Molanorouzi K., Khoo S., Morris T. (2015). Motives for adult participation in physical activity: Type of activity, age, and gender. BMC Public Health.

[6] Prochaska J.O., DiClemente C.C. (1983). Stages and processes of self-change of smoking: Toward an integrative model of change. J. Consult. Clin. Psychol..

[7] Liu K.T., Kueh Y.C., Arifin W.N., Kim Y., Kuan G. (2018). Application of transtheoretical model on behavioral changes, and amount of physical activity among university’s students. Front. Psychol..

[8] Abbaspour S., Farmanbar R., Njafi F., Ghiasvand A.M., Dehghankar L. (2017). Decisional balance and self-efficacy of physical activity among the elderly in Rasht in 2013 based on the transtheoretical model. Electron. Physician.

[9] Bernard P., Romain A.J., Trouillet R., Gernigon C., Nigg C., Ninot G. (2014). Validation of the TTM processes of change measure for physical activity in an adult French sample. Int. J. Behav. Med..

[10] Janis I.L., Mann L. (1977). Decision Making: A Psychological Analysis of Conflict, Choice and Commitment.

[11] Hausenblas H.A., Nigg C.R., Downs D.S., Connaughton D.P. (2002). Perceptions of exercise stages, barrier self-efficacy and decisional balance for middle-level school students. J. Early Adolesc..

[12] Karaca A., Caglar E., Deliceoglu G., Bilgili N. (2016). Physical activity with regard to socio-demographic variables and decisional balance perceptions for exercise among university students. J. Phys. Educ. Sport.

[13] Prochaska J.O., Velicer W.F. (1997). The transtheoretical model of health behavior change. Am. J. Health Promot..

[14] Prochaska J.O., Veliver W.F., Rossi J.S., Goldstein M.G., Marcus B.H., Rokowski W. (1994). Stages of change and decisional balance for twelve problem behaviors. Health Psychol..

[15] Han H., Gabriel K.P., Kohl H.W. (2015). Evaluations of validity and reliability of a transtheoretical model for sedentary behavior among college Students. Am. J. Health Behav..

[16] Zamarripa J., Marentes-Castillo M., Castillo I., Delgado M., Alvarez O. (2017). Decisional balance, motivation and exercise enjoyment in a Mexican population Sample. J. Sport Psychol..

[17] Calfas K.J., Sallis J.F., Lovato C.Y., Campbell J. (1994). Physical activity and its determinants before and after college graduation. Med. Exerc. Nutr. Health.

[18] Marcus B.H., Rakowski W., Rossi J.S. (1992). Assessing motivational readiness and decision making for exercise. Health Psychol..

[19] Plotnikoff R.C., Blanchard C., Hotz S.B., Rhodes R. (2001). Validation of the decisional balance scales in the exercise domain from the transtheoretical model: A longitudinal test. Meas. Phys. Educ. Exerc. Sci..

[20] Rizal H., Hajar M.S., Kueh Y.C., Muhamad A.S., Kuan G. (2019). Confirmatory factor analysis of the Malay- language transtheoretical model of physical activity among Malaysian primary school children. Malays J. Med. Sci..

[21] Kline R.B. (2011). Principles and Practice of Structural Equation Modelling.

[22] Hair J.F., Black W.C., Babin B.J., Anderson R.E. (2010). Multivariate Data Analysis.

[23] Colton T. (1974). Statistics in Medicine.

[24] Liu K.T., Kuan G., Arifin W.N., Kueh Y.C. (2019). Psychometric properties of the self-efficacy scale among undergraduate students in Malaysia. Malays J. Med. Sci..

[25] Sabo A., Kueh Y.C., Kuan G. (2019). Psychometric properties of the Malay version of the self-efficacy for exercise scale. PLoS ONE.

[26] Kline R.B. (2015). Principles and Practice of Structural Equation Modeling.

[27] Blaney C.L., Robbins M.L., Paiva A.L. (2012). Validation of the measures of the transtheoretical model for exercise in an adult African-American sample. Am. J. Health Promot..

